# Supplementary material for: Emerging Antibiotic Resistance Patterns Affect Visual Outcome Treating Acute Endophthalmitis
Source: Antibiotics (Basel). 2022 Jun 23;11(7):843. doi: 10.3390/antibiotics11070843 (PMC9311540; doi:10.3390/antibiotics11070843)
Supplement: Supplementary file 1 [file antibiotics-11-00843-s001.zip › antibiotics-1720081-supplementary.pdf]

Table S1. Intraocular culture results.

Culture results	Anterior Chamber, # (%)	Vitreous, # (%)
Culture negative	127 (78.4)	129 (58.9)
Staphylococcus Epidermidis	7 (4.32)	25 (10.96)
Streptococcus Mitis	4 (2.47)	11 (5.02)
Haemophilus Influenza	6 (3.7)	8 (4.65)
Pseudomonas Aerugenosa	4 (2.47)	5 (2.28)
Alpha-Haemolytic Streptococcus	0 (0)	5 (2.28)
Streptococcus Salivarius	1 (0.62)	4 (1.83)
Enterococcus Faecalis	1 (0.62)	3 (1.37)
Staphylococcus Aureus	2 (1.23)	3 (1.37)
Streptococcus Pneumoniae	1 (0.62)	3 (1.37)
Corynebacterium Tuberculostearicum	0 (0)	2 (0.91)
Moraxella Nonliquefaciens	2 (1.23)	2 (0.91)
Staphylococcus Warneri	1 (0.62)	2 (0.91)
Sphingomonas Paucimobilis	1 (0.62)	2 (0.91)
Gram negative rods	0 (0)	2 (0.91)
Acinetobacter lwoffii	2 (1.23)	1 (0.46)
Streptococcus Acidominimus	1 (0.62)	1 (0.46)
Achromobacter Xylosoxidans	0 (0)	1 (0.46)
Aeromonas Sobria	0 (0)	1 (0.46)
Citrobacter Koseri	0 (0)	1 (0.46)
Enterococcus Cloacae	0 (0)	1 (0.46)
Gram positive cocci	0 (0)	1 (0.46)
Lactobacillus Paracasei	0 (0)	1 (0.46)
Listeria Monocytogenes	0 (0)	1 (0.46)
Neisseria Flavescens	0 (0)	1 (0.46)
Neisseria Meningitis	0 (0)	1 (0.46)
Staphylococcus Lugdunensis	0 (0)	1 (0.46)
Streptococcus Viridans	0 (0)	1 (0.46)
Stenotrophomonas Maltophilia	0 (0)	1 (0.46)
Gram positive rods	1 (0.62)	0 (0)
Micrococcus Luteus	1 (0.62)	0 (0)
